# Pneumocephalus after Tympanomastoidectomy: A Case Presentation

**Published:** 2018-05

**Authors:** Mohammadhossein Baradaranfar, Sedighe Vaziribozorg, Mojtaba Mirzade, Mostafa Salari

**Affiliations:** 1 *Department of Otolaryngology- Head and Neck Surgery* *, Otorhinolaryngology Research Center, Shahid Sadoughi University of Medical Sciences, Yazd, Iran.*; 2 *Faculty of Medicine, Shahid Sadoughi University of Medical Sciences, Yazd, Iran.*

**Keywords:** Mount Fuji sign, Mastoidectomy, Pneumocephalus

## Abstract

**Introduction:**

Pneumocephalus is the presence of air or gas within the cranial cavity. It can occur following otorhinolaryngological procedures. A small pneumocephalus spontaneously heals without any treatment. In severe cases, conservative therapy includes a 30-degree head elevation, avoidance of the Valsalva maneuver, analgesics, osmotic diuretics, and oxygen therapy.

**Case Report:**

A 56-year-old woman was referred to the emergency department due to a severe headache in the frontal area for 2 days before admission. The patient experienced nausea and vomiting in the morning and had no history of seizures or decreased consciousness. Examination of neurological symptoms was completely normal and showed no symptoms of meningeal irritation. In terms of past history, the patient had undergone tympanomastoidectomy surgery and resection of the cholesteatoma 1 week previously. The Mount Fuji sign was found on the brain computed tomography (CT) scan of the patient. Treatments such as CBR (complete bed rest), 30-degree head elevation, anti-fever, analgesics and oxygen therapy, along with anti-compulsive drug (phenytoin), were prescribed. At the end of 5 days, the patient's pneumocephalus was resolved completely.

**Conclusion:**

Pneumocephalus should be considered a post-operative complication of tympanomastoidectomy. In most cases, pneumocephalus responds to conservative therapy. Supplemental oxygen increases the rate of absorption of pneumocephalus. Serial imaging is needed to ensure gradual reduction of the pneumocephalus**.**

## Introduction

The presence of air or gas within the cranial cavity is called pneumocephalus**.** It usually occurs after head and facial trauma (fracture on the base of the skull), tumor invasion of the skull base, middle ear infection, following neurosurgery or otorhinolaryngological procedures (thoracotomy,mastoid surgery), or encephalocele(1), and can rarely occur spontaneously due to congenital defects of the tegmen tympani (2). 

It presents with headaches, nausea and vomiting, seizures, dizziness, and a depressed neurological status (3). The term “tension pneumocephalus” was coined by Wolf and Aktor to describe the situation when air inside the brain causes an increase in intracranial pressure (4,5).

The presence of air inside the cranium can be acute (lasting less than 72 hours) or chronic (more than 72 hours) (6,7). The volume of pneumocephalus gas also varies from 25 ml to 65 ml, but a volume of 25 ml can lead to tension pneumocephalus (8-10). 

Significant radiologic symptoms of tension pneumocephalus are shown on a computed tomography (CT) scan as the "Mount Fuji sign", which was first described by Ishiwata in 1988. The air inside the cranium separates the anterior space of the two hemispheres from the frontal tip, imitating a state similar to that of the famous Fuji mountain in Japan (11). Another symptom of tension pneumocephalus on the CT scan is the “Peaking sign”, which exerts pressure on the anterolateral and lateral lobe of the frontal lobe, but does not cause the lobes to break apart (12).

## Case Report

A 56-year-old woman was referred to the emergency department due to a severe headache in the frontal area for 2 days before admission. The patient had nausea and vomiting in the morning but had no history of seizures or decreased consciousness. The examination of neurological symptoms was completely normal and showed no symptoms of meningeal irritation. The patient's vital signs were recorded as follows: blood pressure (BP)=130/85 mmHg, heart rate (HR)= 86/min, and temperature (T)=37.3 °C. In her past history, the patient had undergonetympano- mastoidectomy surgery and resection of the cholesteatoma 1 week earlier, and a canalwall down mastoidectomy (CWD) was performed due to right ear cholesteatoma. According to the information obtained from the previous surgeon, microtrauma was inflicted in the dural plate during surgery, but since no significant cerebrospinal fluid (CSF) leak occurred during the procedure, reconstruction was not necessary. After admission, examination and initial evaluation, the Mount Fuji sign was found on the brain CT scan of the patient, but no evidence of brain abscess or intracranial hemorrhage. The patient was immediately admitted to the intensive care unit (ICU).

Initial treatments such as CBR, 30-degree head elevation, anti-fever therapy, analgesics and oxygen therapy, along with anti-compulsive drug (phenytoin), were prescribed. The patient did not undergo surgery due to a lack of neurological symptoms and a decreased level of consciousness or seizures. However, in the ICU, the patient was maintained under regular and continuous monitoring of vital and neurological signs and level of consciousness. The day after admission, the patient’s headache had completely resolved. The patient was admitted to the ICU for 5 days and was monitored for volume ofpneumocephalus every day with a CT scan. At the end of 5 days, the patient's pneumocephalus was resolved completely, and she was transferred to the ward. The patient was discharged after complete recovery.

## Discussion

Pneumocephalus can be caused by trauma (basal skull fractures, paranasal sinuses fractures, and open cranial convexity fractures with dural laceration), neurosurgical operations, intracranial pressure (ICP) monitoring, trans-sphenoidal or endoscopic sinus surgery, ear, nose, and throat (ENT) operations (paranasal sinuses surgery), lumbar punctures, barotraumas, tumors, central nervous system infections caused by gas-producing microorganisms, nitrous oxide, congenital skull and tegmen tympani defects, spinal anesthesia, positive pressure ventilation, and hyperbaric oxygen therapy, as well as spontaneously (13). Patients undergoing neurosurgical or ocular surgery are at risk of pneumocephalus and tension pneumocephalus. Although tension pneumocephalus after surgery is very rare, even minor damage to the dural plate during ear surgery can allow air to enter the cranial cavity. In general, there are two possible mechanisms for this state: 1) The "Ball valve" effect where air can enter the cranial cavity due to CSF leak but cannot be removed, and consequently tension pneumocephalus occurs; 2) Inverted soda battle theory where the fistula or drainage out of the CSF causes negative pressure and causes air to replace the CSF (14).

These mechanisms, along with postural change and infection by gas-forming organisms, may be responsible for the accumulation of intracranial air, leading to the formation of tension pneumocephalus. ICP increases in response to the mass effect of the air. If pressure is not relieved, the situation can rapidly become fatal. Pneumocephalus is asymptomatic unless it is under tension. There may be a latent period before symptoms of increased ICP manifest with or without the features of meningitis (15).

Diagnosis of pneumocephalus is based on otologic examination and history. Pneumocephalus should be considered in patients with a history of mastoid surgery, trauma, CSF otorrhea, headache, seizure, and meningismus (16). Clinical and radiological symptoms, including CT scan findings, are very effective in diagnosis (14). Pneumocephalus may be asymptomatic or may involve symptoms such as headache, rhinorrhea, or otorrhea and seizures (17). 

Symptoms of tension pneumocephalus include the mass effect and a decrease in consciousness and neurological disorders. The most significant radiologic symptom of tension pneumocephalus in a CT scan is the “Mount Fuji sign”.

A small pneumocephalus spontaneously heals without any treatment. In severe cases, conservative therapy includes a 30-degree head elevation and avoidance of Valsalva maneuvers, such as sneezing and coughing, as well as analgesics and anti-fever therapy, osmotic diuretics and oxygen therapy. By administering these treatments, up to 85% of cases are treated within 2 to 3 days (10). Emergency decompression is performed in cases with clinical neurologic defects and is carried out by bure hole and drill or even needle (18).

**Fig1:**
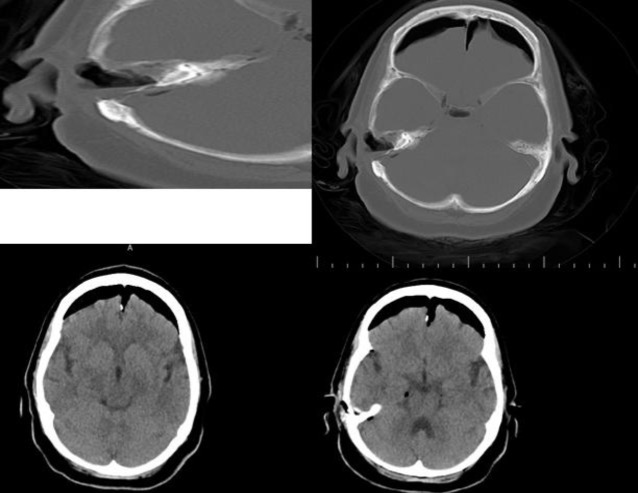
First day after pneumocephalus

**Fig 2:**
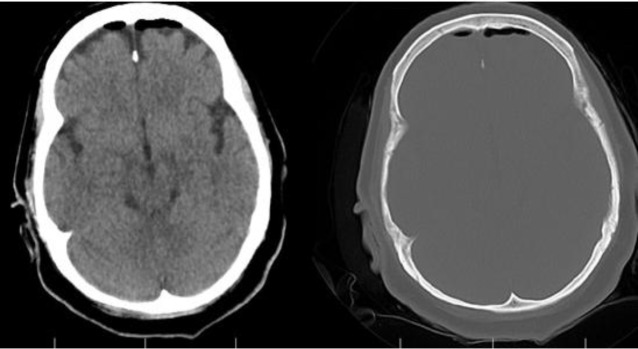
Third day afterpneumocephalus

**Fig 3:**
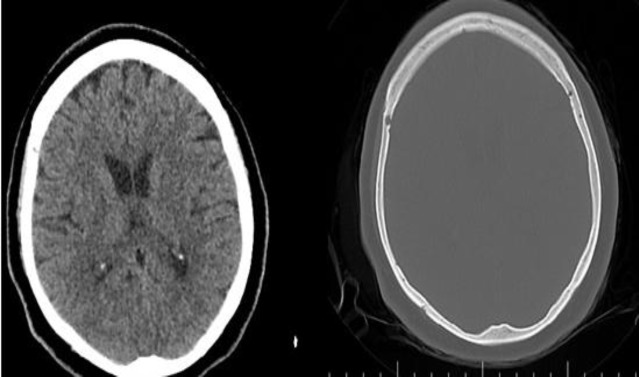
Fifth day afterpneumocephalus

Our patient had the “Mount Fuji sign” on her CT scan, but had no neurological symptoms and a decrease in consciousness, and had not yet entered the tension pneumocephalus stage.According to the information obtained from the previous surgeon, it is likely that a microtrauma to the dural plate, without repair, had led to the pneumocephalus. The patient was admitted to the ICU and was treated with oxygen therapy and conservative therapy. This was followed up by a brain CT scan, and the patient was discharged after a complete recovery (Figs.1- 3).

## Conclusion

Pneumocephalus should be considered as a post-operative complication ofthe very common otolaryngologicalprocedure, tympano- mastoidectomy. Even minor air collection in the cranial cavity carries a risk of transformation into tension pneumocephalus in the case of valve mechanism development. CT and magnetic resonance imaging (MRI) scans are required for an accurate diagnosis. In most cases, pneumocephalus responds to conservative therapy. 

Supplemental oxygen increases the rate of absorption of pneumocephalus. Serial imaging is needed to ensure gradual reduction of the pneumocephalus**. **

It is sensible that at discharge, all patients who have undergone otolaryngological procedures should be educated about the signs and symptoms that can predict the onset of intracranial complications.
